# Glutamate receptor activation triggers OPA1 release and induces apoptotic cell death in ischemic rat retina

**Published:** 2008-12-31

**Authors:** Won-Kyu Ju, James D. Lindsey, Mila Angert, Ankur Patel, Robert N. Weinreb

**Affiliations:** Hamilton Glaucoma Center and Department of Ophthalmology, University of California San Diego, La Jolla, CA

## Abstract

**Purpose:**

Glutamate receptor activation-induced excitotoxicity has been hypothesized to cause retinal ganglion cell (RGC) death in glaucoma and to link mitochondrial dysfunction in both acute and chronic neurodegenerative disorders. However, the relationships among elevated intraocular pressure (IOP), glutamate receptor-mediated excitotoxicity, and mitochondrial dysfunction in glaucoma remains unknown. The goal of this study was to determine whether the N- methyl D-aspartate (NMDA) glutamate receptor antagonist MK801 can block optic atrophy 1 (OPA1*)* release and subsequent apoptotic cell death, as well as whether acute IOP elevation triggers OPA1 release and alters *OPA1* gene and protein expression in the rat retina after ischemia.

**Methods:**

Sprague Dawley rats received injections of MK801 (10 mg/kg) or vehicle and then transient retinal ischemia was induced by acute IOP elevation. Following subcellular fractionation, changes in cytoplasmic and mitochondrial OPA1 were assessed by western blot analysis. Also, the expression of *OPA1* mRNA was measured by Taqman qPCR, the distribution of OPA1 protein was assessed by immunohistochemistry, and apoptotic cell death was assessed by terminal deoxynucleotidyl transferase dUTP nick end labeling (TUNEL) staining.

**Results:**

The ~65 and 90 kDa isoforms of OPA1 were increased in the cytosol in the rat retina at 6 h and at 12 h, but only the 90 kDa isoform of OPA1 was decreased at 12 h after ischemia induced by acute IOP elevation. This suggests that ischemic insult induced OPA1 release from the mitochondria in retinas. Pretreatment with MK801 blocked this effect and significantly increased OPA1 immunoreactivity in the inner retinal layers, as well as *OPA1* gene expression and total protein expression in retinas at 12 h after ischemia. Further, pretreatment with MK801 prevented apoptotic cell death in retinas at 12 h after ischemia. Following acute IOP elevation, *Bcl-2* mRNA expression in retinas was decreased at 3 h and 6 h but increased at 12 h and 24 h. In contrast, *Bax* mRNA expression in these retinas was increased in the first 12 h and then plateaued. Moreover, pretreatment with MK801 increased *Bcl-2* mRNA expression, but did not alter the course of *Bax* mRNA expression.

**Conclusions:**

These results indicate that OPA1 release from mitochondria triggered by acute IOP elevation is inhibited by blockade of glutamate receptor activation. Because this effect was accompanied by increases of *Bcl-2* expression, no changes of *Bax* expression, and blockade of apoptosis, these findings indicate that glutamate receptor activation following acute IOP elevation may lead to a distinct mitochondria-mediated cell death pathway in ischemic retina. These results support further studies to determine whether ischemia-induced OPA1 release may be an important component of the biochemical cascade leading to pressure-related ischemic damage in glaucomatous retina.

## Introduction

Elevated intraocular pressure (IOP) is an important risk factor for optic nerve damage in glaucoma [1]. However, the precise pathophysiological relationship between elevated IOP and retinal ganglion cell (RGC) death remains poorly understood. It has been hypothesized that glutamate receptor activation may contribute to RGC death in glaucoma [2]. In addition, glutamate receptor activation-induced excitotoxicity has been linked to mitochondrial dysfunction in both acute and chronic neurodegenerative disorders [3-6]. However, the relationship among elevated IOP, glutamate excitotoxicity, and mitochondrial dysfunction in glaucoma remains unknown. Recently, we reported that moderately elevated hydrostatic pressure could induce abnormal cristae depletion, cytochrome C release, cellular ATP reduction, and translocation of dynamin-related protein-1 (Drp-1) in differentiated RGC-5 cells [7]. Further, we also found that elevated hydrostatic pressure triggers release of optic atrophy type 1 protein (OPA1) and cytochrome C, and induces subsequent apoptotic cell death in differentiated RGC-5 cells [8]. These observations raise the possibility that pressure-induced mitochondrial dysfunction may contribute to RGC death in glaucoma.

In healthy cells, mitochondria are autonomous and morphologically dynamic organelles that structurally reflect a precise balance of ongoing fission and fusion within a cell [9-11]. This balance is regulated by a family of dynamin-related GTPases that exert opposing effects. Drp-1 regulates mitochondrial fission, while OPA1, the human ortholog of Mgm1p/Msp1p, and the mitofusins are required for mitochondria fusion [10,12]. *OPA1* mRNA is transcribed from nuclear DNA and new OPA1 protein is then translocated to the inner membrane of mitochondria [13,14]. Of particular interest, mutations in *OPA1* are linked with neurodegenerative diseases in human and can cause autosomal dominant optic atrophy (ADOA), the most common form of hereditary optic neuropathy [15,16]. Retinal OPA1 is expressed in the soma and axons of the RGCs as well as in horizontal cells [17-19]. Although the specific functional role of OPA1 in these cells remains unknown, it has been shown that down-regulation of *OPA1* causes mitochondrial fission, leading to cytochrome C release and apoptosis in HeLa cells, as well as induces aggregation of the mitochondrial network in purified RGCs [20-23]. Proteolytic processing of OPA1 has been observed during mitochondrial fission, although its significance remains poorly investigated [24-27]. Also, OPA1 release during mitochondrial fission contributes to apoptotic cell death [22,26]. Nevertheless, it is unknown whether acute IOP elevation can directly alter OPA1 expression and distribution in the mammalian retina.

Thus, we evaluated whether the N- methyl D-aspartate (NMDA) glutamate receptor antagonist MK801 can block OPA1 release and subsequent apoptotic cell death as well as whether acute IOP elevation triggers OPA1 release and alters *OPA1* gene and protein expression in the rat retina after ischemia. Also, we compared the progression of these changes with changes in *Bcl-2* and *Bax* expression.

## Methods

### Transient retinal ischemia

All procedures were in compliance with the National Institute of Health Guide for the Care and Use of Laboratory Animals and the ARVO Statement on the Use of Animals in Ophthalmic Research. Sprague-Dawley female rats, 3 months of age (200–250 g in weight) were anesthetized with ketamine (100 mg/kg) and xylazine (20 mg/kg, i.p. injection). A cannula was inserted into the anterior chamber that was connected by flexible tubing to a reservoir. By raising the reservoir, IOP was elevated above systolic blood pressure (100–120 mmHg) for 60 min. Animals were allowed to recover for 3 to 24 h.

### Pharmacological treatment

Two groups of rats were studied following unilateral transient retinal ischemia: a group treated with vehicle (0.9% saline, i.p. injection) and a group treated with MK801 (10 mg/kg in 0.9% saline; i.p. injection; n=5 animals/group; Sigma, St. Louis, MO) given 30 min before ischemia.

### Tissue preparations

The light-adapted rats were anesthetized with isoflurane followed by an i.p. injection of ketamine/xylazine as above. Both eyes were enucleated and then the rats were killed by CO_2_ inhalation. The retinas were dissected from the choroid and fixed in the 4% paraformaldehyde in 0.1 M phosphate buffer (PB, pH 7.4) for 2 h at 4 °C. Retinas were dehydrated through graded ethanols and then embedded in polyester wax [18].

### Western blot analysis

The retinas were homogenized in a glass-teflon Potter homogenizer in lysis buffer (20 mM Hepes, pH 7.5, 10 mM KCl, 1.5 mM MgCl_2_, 1 mM EDTA, 1 mM EGTA, 1 mM DTT, 0.5% CHAPS, complete protease inhibitors; Roche Biochemicals, Indianapolis, IN). Each sample (10 µg) was separated by PAGE and electro-transferred to Polyvinylidene Fluoride (PVDF) membranes. The membrane was blocked with 5% nonfat dry milk and 0.05% in Tween-20 in phosphate buffer saline (PBS), incubated with monoclonal mouse anti-OPA1 antibody (H-300/1:1000; BD Transduction Laboratories, San Diego, CA) or monoclonal mouse anti-actin antibody (Ab-1/1:3,000; Calbiochem, La Jolla, CA), rinsed with 0.05% Tween-20 in PBS, incubated with peroxidase-conjugated goat anti-mouse IgG (1:2,000; Bio-Rad, Hercules, CA), or goat anti-rabbit IgM (1:5,000; Calbiochem), and developed using chemiluminescence detection (ECL Plus; GE Healthcare Bio-Sciences, Picataway, NJ). Images were analyzed by digital fluorescence imager (Storm 860; GE Healthcare Bio-Sciences) and band densities were normalized using actin as cytosolic fraction calibrator and Voltage-dependent Anion Channel (VDAC) as mitochondrial fraction calibrator with ImageQuant TL (GE Healthcare Bio-Sciences).

To assess the subcellular distribution of OPA1, the cytosolic and mitochondrial fractions were isolated from retinas by differential centrifugation (Mitochondrial Isolation Kit; Pierce, Rockford, IL) and western blot analysis was performed as above. Equal loading was confirmed by reprobing with actin as above or with polyclonal rabbit anti-VDAC antibody (Ab-5/1:1,000, Calbiochem).

### Taqman quantitative polymerase chain reaction

Total RNA from retinas was extracted with Trizol (Invitrogen, Carlsbad, CA), purified on RNeasy mini columns (Qiagen, Valencia, CA), and treated with RNase-free DNase I (Qiagen). *OPA1*, *Bax,* and *Bcl-2* gene expression were measured by Taqman quantitative PCR (MX3000P, Stratagene, La Jolla, CA) using 50 ng of cDNA from retinas and 2X Taqman universal PCR master mix (Applied Biosystems, Foster City, CA) with a one-step program (95 °C for 10 min, 95 °C for 30 s, and 60 °C for 1 min for 50 cycles). Primers for *OPA1*, *Bax*, *Bcl-2*, and *GAPDH*, as well as Taqman probe for *GAPDH* were designed using Primer Express 2.0 software (Applied Biosystems), obtained from Roche Diagnostics (Mannheim, Germany; Table 1), and the optimal concentrations for probe and primers were determined using heart tissue. Duplicate samples without cDNA (no-template control) showed no contaminating DNA. Standard curves were constructed using nine twofold dilutions (50 ng-0.195 ng) for both the targets (*OPA1, Bax* and *Bcl-2*) and the endogenous reference (*GAPDH*). The samples were run in triplicates for target and endogenous *GAPDH* control.

**Table 1:** Primer and probe sequences for *OPA1, Bax, Bcl-2,* and *GAPDH* for Taqman quantitative PCR^a^.

**Gene (GenBank)**	**Type**	**Sequences (5′–3′)**
*OPA1* rat (NM_133585)	Forward	CAGCTGGCAGAAGATCTCAAG
	Reverse	CATGAGCAGGATTTTGACACC
	Probe	Universal Probe Library probe #2
		Cat. # 04684982001
*Bax* rat (NM_017059)	Forward	GTGAGCGGCTGCTTGTCT
	Reverse	GTGAGCGGCTGC-TTGTCT
	Probe	Universal Probe Library probe #83
		Cat. # 04689062001
*Bcl-2* rat (NM_016993)	Forward	GGGATGCCTTTGTGGAACT
	Reverse	CTGAGCAGCGTCTTCAGAGA
	Probe	Universal Probe Library probe #2
		Cat. # 04684982001
*GAPDH* rat (X02231)	Forward	GAACATCATCCCTGCATCCA
	Reverse	CCAGTGAGCTTCCCGTTCA
	Probe	CTTGCCCACAGCCTTGGCAGC

^a^Taqman probe containing 5′ reporter FAM and 3′ quencher BHQ-1 dyes.

### Immunohistochemical analysis

Immunohistochemical staining 7 μm wax section of full thickness retina was done by the Tyramide Signal Amplication Kit (Molecular Probes, Eugene, OR) as described below [18]. To increase the sensitivity of OPA1 immunohistochemistry, sections exposed to primary and secondary antibodies were incubated with solutions from the Tyramide Signal Amplification Kit (Molecular Probes). Tissues were permeabilized with 0.1% Triton X-100 in PBS, incubated with quenching buffer (Amplication buffer and 0.0015% H_2_O_2_) for 1 h at room temperature, blocked with 1% BSA/PBS, and then incubated with antibody against OPA1 (provided by Drs. Misaka and Kubo, University of Tokyo, 1:100) for 16 h at 4 °C. After several washing, tissues were incubated with peroxidase-conjugated goat anti-rabbit IgG for 1 h at room temperature, washed, and incubated with tyramide working solution for 10 min at room temperature.

### TUNEL staining

Sections were incubated with proteinase K (10 µg/ml, 10 mM Tris, pH 7.4–8.0) for 10 min at 37 °C. After rinsing in PBS, the sections were incubated with terminal deoxynucleotidyl transferase plus nucleotide mixture in reaction buffer for 60 min at 37 °C (In situ Cell Death Detection kit, Roche Diagnostics).

### Statistical analysis

Experiments presented were repeated at least three times with triplicate samples. The data are presented as the mean±SD. Comparison of two experimental conditions was evaluated using the unpaired Student’s *t*-test. A p<0.05 was considered to be statistically significant.

## Results

### Acute intraocular pressure elevation induces OPA1 release from mitochondria in the rat retina

**Figure 1:**
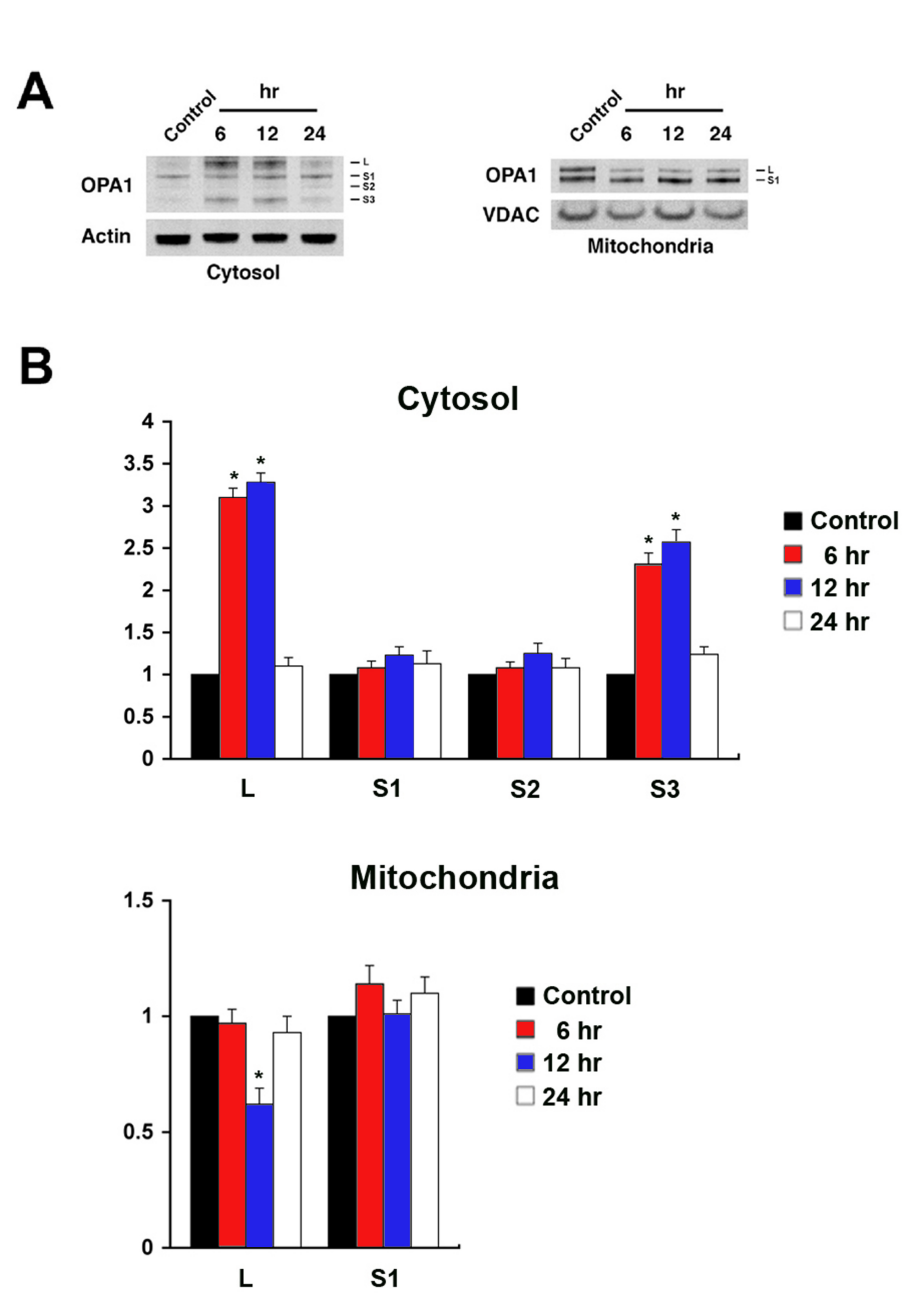
Acute IOP elevation induces OPA1 release in ischemic rat retina. **A**: The OPA1 protein bands observed at 80 kDa in cytosolic fraction and at 80 and 90 kDa forms in mitochondrial fraction of normal retina. Following ischemia, there were at least four major isoforms of OPA1 protein bands (90:L, 80:S1, ~75:S2, and ~65 kDa:S3) in the cytosolic fraction. **B**: Relative intensity of chemiluminescence for each protein band was normalized using actin as cytosolic fraction calibrator and VDAC as mitochondrial fraction calibrator. The isoforms of OPA1 protein (L and S3) were significantly increased in the cytosolic fraction. However, the isoform of OPA1 protein (L) was significantly decreased at 12 h but the isoform of OPA1 protein (S1) was not changed in the mitochondrial fraction at 6–24 h after ischemia. Error bars represent the standard deviation (*p<0.05 by Student’s *t*-test, n=3).

The OPA1 antibody recognized a major band (80 kDa) in the cytosolic fraction and two major bands (90 and 80 kDa) in the mitochondrial fraction of the normal rat retina. After ischemia, at least 4 major isoforms of OPA1 (90 kDa:L, 80 kDa:S1, ~75 kDa:S2, and ~65 kDa:S3) were observed in the cytosolic fraction. In contrast, only 2 major isoforms of OPA1 (L and S1) were observed in the mitochondrial fraction (Figure 1A). Increased L and S3 isoforms were prominent in the cytosolic fraction at 6 h and at 12 h after ischemia but there were no changes in the S1 and S2 isoforms. In the mitochondrial fraction from ischemic retina, L isoform OPA1 was significantly decreased at 12 h but there was no significant change in S1 isoform OPA1 (Figure 1B).

### Blockade of glutamate receptor activation increases *OPA1* gene and protein expression in the rat retina following transient ischemia

**Figure 2:**
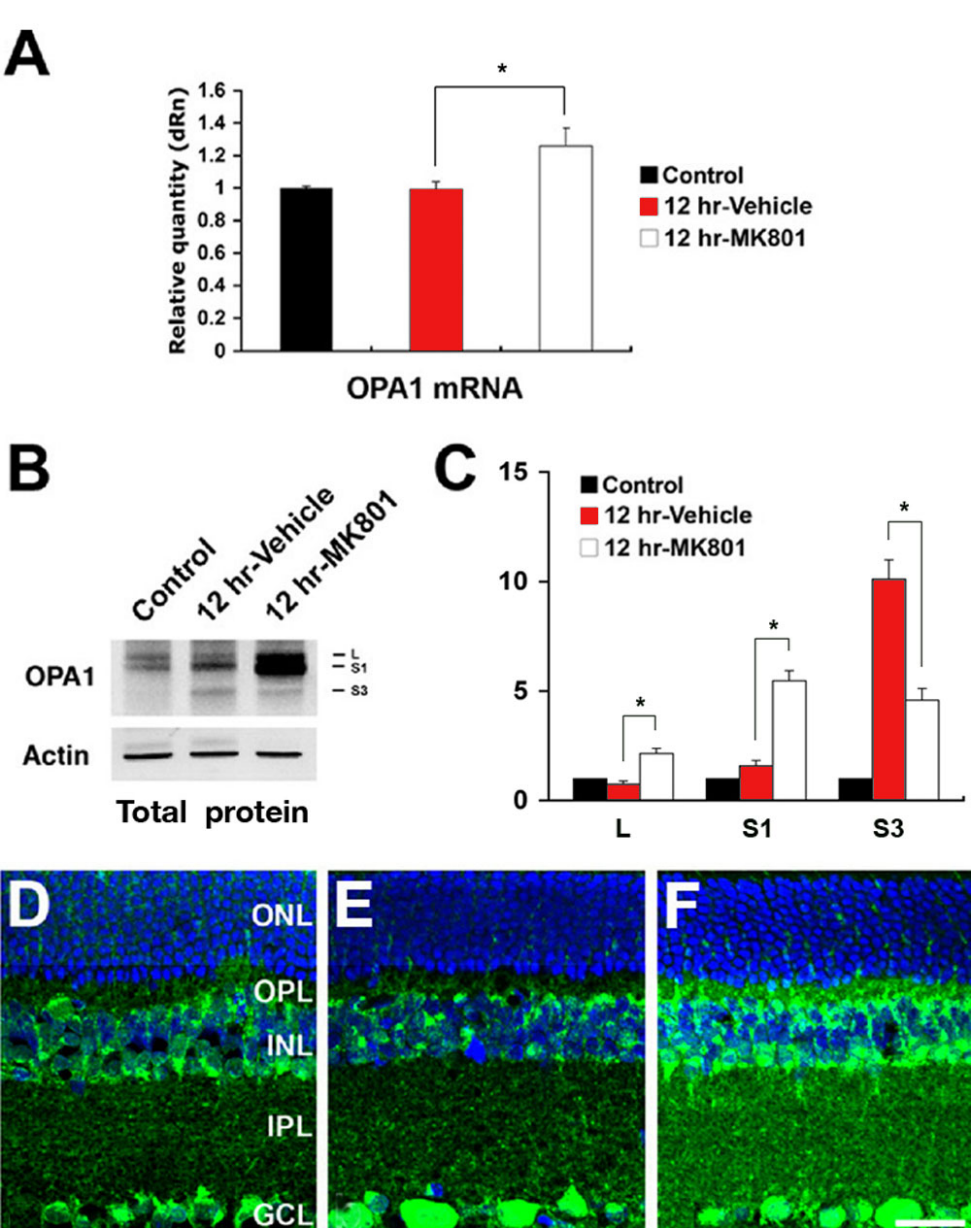
Blockade of glutamate receptor activation increases *OPA1* gene and protein expression in ischemic rat retina. **A:** *OPA1* gene expression was not changed in vehicle-pretreated ischemic retinas but increased in MK801-pretreated ischemic retinas at 12 h. **B:** The total isoformes of OPA1 protein bands (80–90 kDa) in the normal rat retina were increased after ischemia, and had a small released fragment of OPA1 (~65 kDa:S3). Pretreatment with MK801 induced a larger increase of total L and S1 isoforms of OPA1 protein bands but decrease of total S3 isoform of OPA1 protein band. **C:** Relative intensity of chemiluminescence for each protein band was normalized using actin as cytosolic fraction calibrator. Error bars represent the standard deviation (*p<0.05 by Student’s *t*-test, n=3). **D-F:** OPA1 immunoreactivity in normal retina (**D**), retina of vehicle pre-treated rats at 12 h after ischemia (**E**), and retina of MK801 pre-treated rats at 12 h after ischemia (**F**). Abbreviations: ONL represents outer nuclear layer; OPL represents outer plexiform layer; INL represents inner nuclear layer; IPL represents inner plexiform layer; GCL represents ganglion cell layer. The scale bar represents 20 μm (**D-F**).

**Figure 3:**
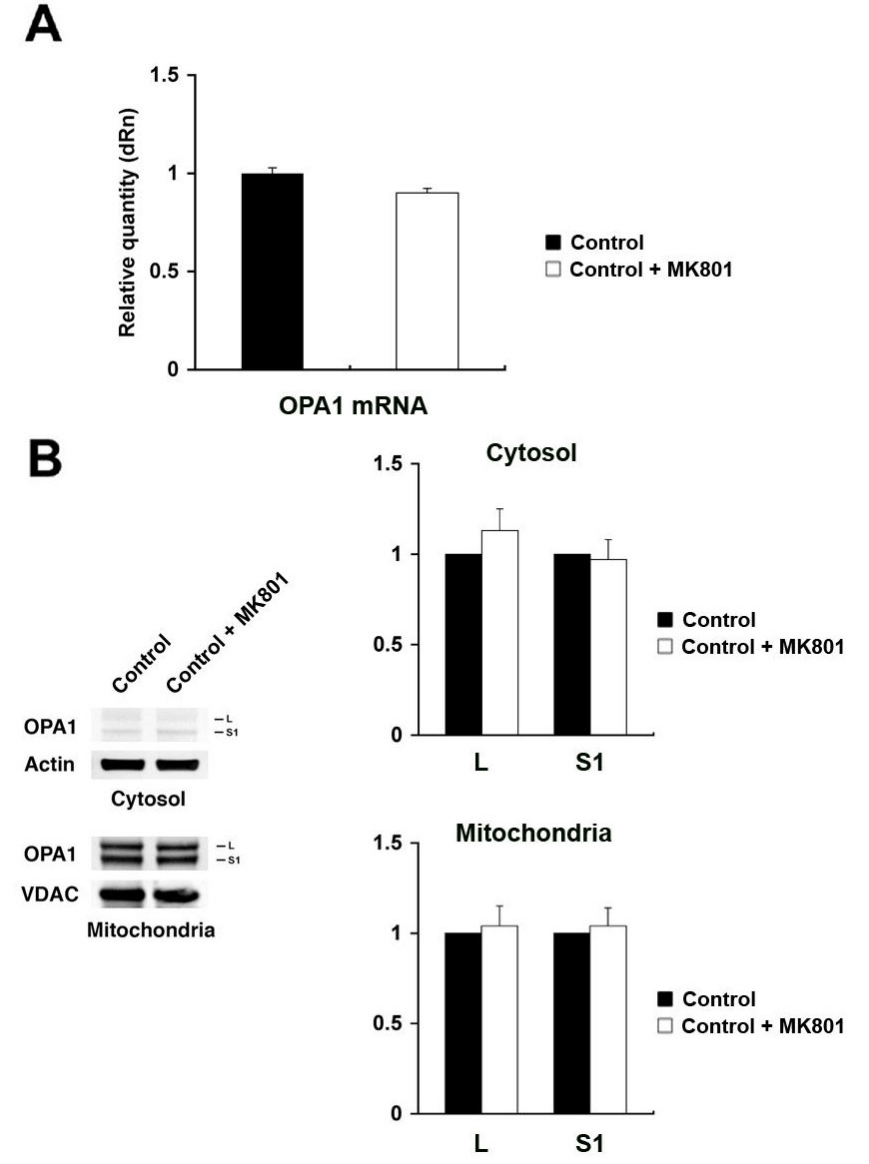
The effect of MK801 in the normal rat retina. **A**: *OPA1* gene expression was not changed in MK801-pretreated normal control retinas compared to non-treated normal control retinas. **B:** The OPA1 protein bands observed at 80 and 90 kDa forms in cytosolic and mitochondrial fractions in both non-treated and MK801-treated normal control retina. Relative intensity of chemiluminescence for each protein band was normalized using actin as cytosolic fraction calibrator and VDAC as mitochondrial fraction calibrator. Note that there were no significant changes between non-treated and MK801-treated normal control retina. Error bars represent the standard deviation.

*OPA1* mRNA expression did not differ in vehicle-pretreated retina after ischemia, compared to normal retina. However, pretreatment with MK801 significantly increased *OPA1* mRNA expression by 26.0±1.1% in retina after ischemia (Figure 2A). Interestingly, pretreatment with MK801 significantly increased total L and S1 isoforms of OPA1 protein at 12 h of ischemic retina but significantly decreased total S3 isoform of OPA1 protein (Figure 2B,C). OPA1 immunoreactivity was localized in the ganglion cell layer (GCL), inner plexiform layer (IPL), inner nuclear layer (INL), and outer plexiform layer (OPL) in the normal rat retina as previously reported (Figure 2D) [18]. In ischemic rat retina pretreated with vehicle, OPA1 immunoreactivity in the GCL, IPL, INL, and OPL did not show significant changes at 12 h (Figure 2E). In contrast, pretreatment with MK801 significantly increased OPA1 immunoreactivity in the IPL, INL, and OPL at 12 h after ischemia (Figure 2F). In addition, we found that there were no significant changes of both *OPA1* mRNA and protein expression level between two control conditions (non-pressurized retina and non-pressurized retina plus MK801; Figure 3).

### Blockade of glutamate receptor activation significantly prevents OPA1 release in the rat retina following transient ischemia

**Figure 4:**
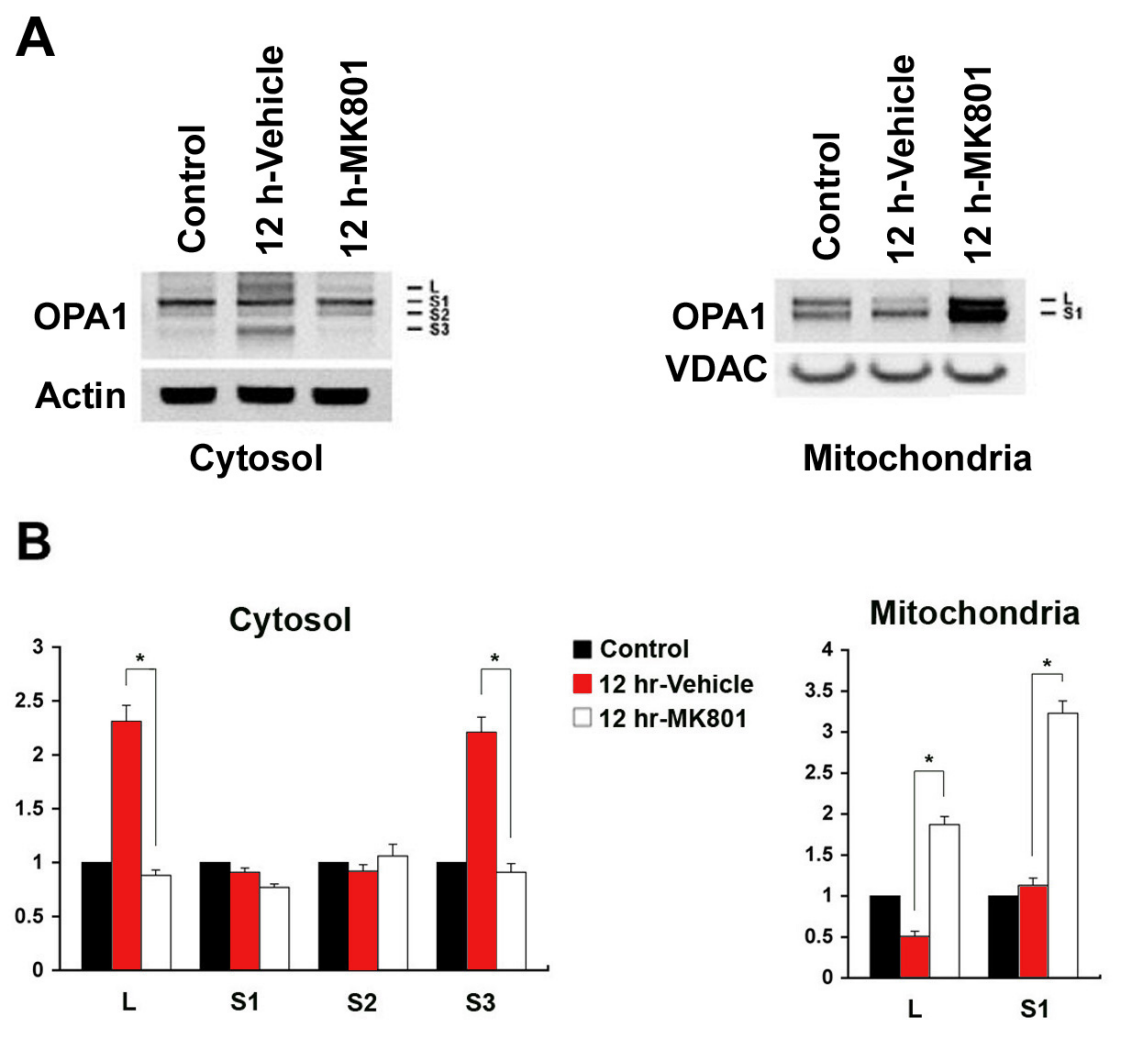
Blockade of glutamate receptor activation blocks OPA1 release in ischemic rat retina. **A**: The OPA1 protein bands observed at 80 kDa in cytosolic fraction and at 80 and 90 kDa forms in mitochondrial fraction of normal retina. Following ischemia, there were at least 4 major isoforms of OPA1 protein bands (90:L, 80:S1, ~75:S2, and ~65 kDa:S3) in the cytosolic fraction. **B**: Relative intensity of chemiluminescence for each protein band was normalized using actin as cytosolic fraction calibrator and VDAC as mitochondrial fraction calibrator. The isoforms of OPA1 protein (L and S3) were significantly increased in the cytosolic fraction and decreased in the mitochondrial fraction at 12 h after ischemia. Pretreatment with MK801 significantly blocks OPA1 release to the cytosol and increased OPA1 protein in the mitochondria at 12 h following ischemia. Error bars represent the standard deviation (*p<0.05 by Student’s *t*-test, n=3).

In ischemic retina pretreated with vehicle, the L and S3 isoforms of OPA1 were significantly increased in the cytosolic fraction (Figure 4A,B). In contrast, the L isoform was significantly decreased in the mitochondrial fraction (Figure 4A,B). Pretreatment with MK801 blocked all of these ischemia-induced changes in cytosolic OPA1. Moreover, pretreatment with MK801 significantly increased both L and S1 isoforms OPA1 in the mitochondrial fraction (Figure 4A,B).

### Blockade of glutamate receptor activation inhibits apoptotic cell death, and increased *Bcl-2* expression but did not alter *Bax* expression

**Figure 5:**
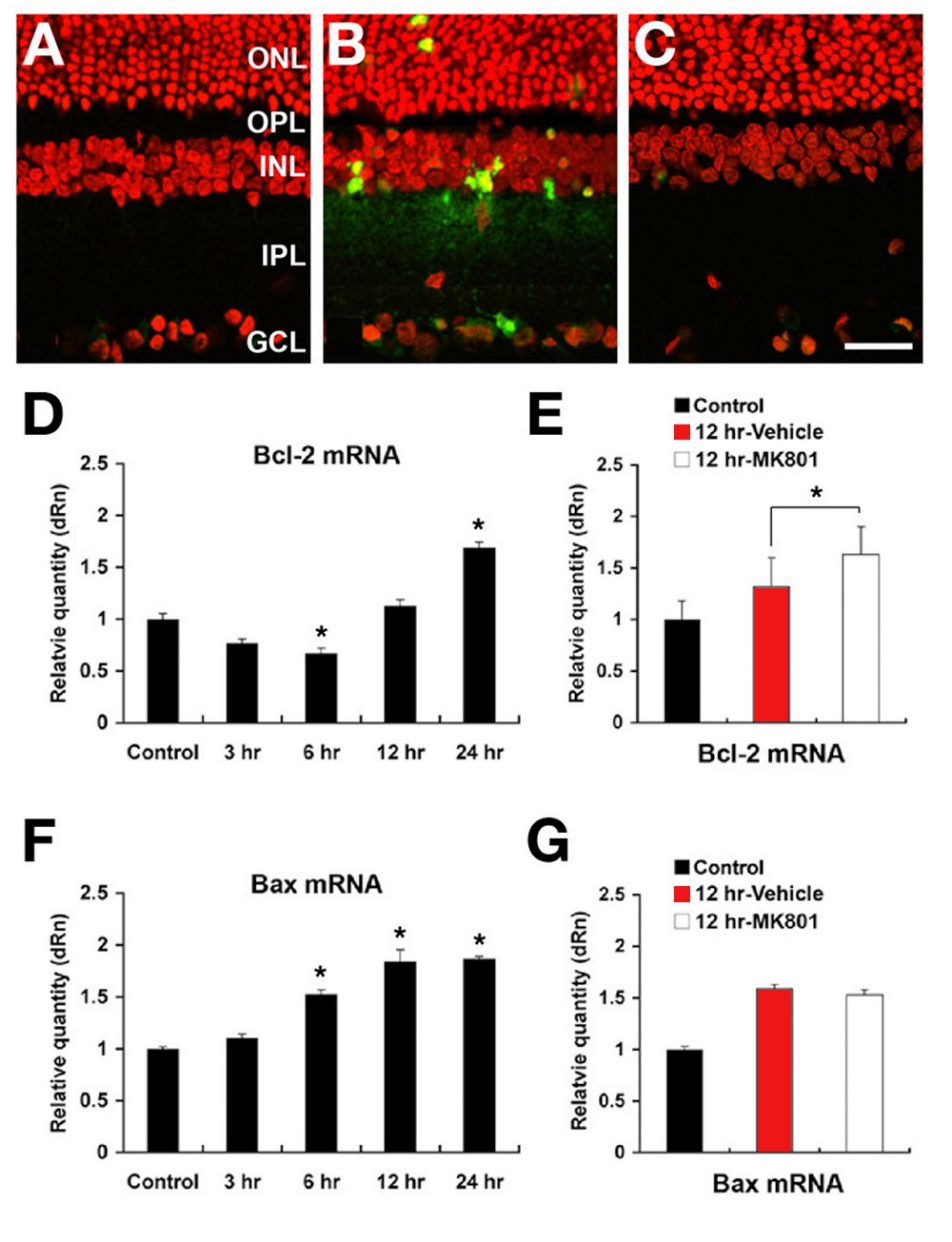
Blockade of glutamate receptor activation blocks apoptotic cell death and induces increase of *Bcl-2* expression in ischemic rat retina. **A**: There were no TUNEL-positive cells in the normal retina. **B**: In ischemic retina of rats receiving vehicle, apoptotic cell death was present in the ONL, INL,and GCL. **C**: MK801 treatment before ischemia blocked apoptotic cell death in all layers. **D**: *Bcl-2* mRNA significantly decreased at 6 h but increased at 24 h, compared to normal retinas. **E**: *Bcl-2* mRNA following ischemia was significantly increase at 12 h by pretreatment with MK801, compared to the vehicle-pretreated retinas. **F**: *Bax* mRNA was significantly increased from 6 to 12 h, compared to normal retina. **G**: No *Bax* expression following ischemia was shown at 12 h by pretreatment with MK801, compared to the vehicle-pretreated retinas. Error bars represent the standard deviation (*p<0.05 by Student’s *t*-test, n=3). Abbreviations: ONL represents outer nuclear layer; OPL represents outer plexiform layer; INL represents inner nuclear layer; IPL represents inner plexiform layer; GCL represents ganglion cell layer. The scale bar represents 20 μm (**A-C**).

In normal retina, there were no TUNEL-positive cells (Figure 5A). Ischemic insult by acute IOP elevation induced apoptotic cell death in the GCL, INL, and ONL at 12 h (Figure 5B). In contrast, pretreatment with MK801 blocked this apoptosis in all layers following ischemia (Figure 5C). *Bcl-2* mRNA was decreased in early phase (3 h and 6 h) but increased in delayed phase (12 h and 24 h) in vehicle-pretreated ischemic retina, compared to normal retina (Figure 5D). In addition, pretreatment with MK801 significantly increased *Bcl-2* mRNA expression by 31.0±2.7% at 12 h (Figure 5E), compared to vehicle-pretreated ischemic retina. *Bax* mRNA expression was increased earlier than *Bcl-2* and plateaued by 12 h in vehicle-pretreated ischemic retina (Figure 5F). Pretreatment with MK801 did not significantly change *Bax* mRNA expression at 12 h (Figure 5G), compared to vehicle-pretreated ischemic retina.

## Discussion

Our results demonstrate that (1) ischemic insult by acute IOP elevation triggers OPA1 release in the early neurodegenerative events (within 12 h), (2) blockade of glutamate receptor activation prevents OPA1 release and apoptotic cell death as well as increases *OPA1* gene and total protein expression at 12 h after ischemia, and (3) blockade of glutamate receptor activation induces increases of *Bcl-2* expression but no changes of *Bax* expression at 12 h after ischemia. These findings indicate that glutamate receptor activation following acute IOP elevation may lead to a distinct mitochondria-mediated cell death pathway in ischemic retina. Further, this ischemia-induced OPA1 release may be an important component of the biochemical cascade leading to pressure-related ischemic damage in glaucomatous retina.

Emerging evidence indicates that mitochondrial morphology and dynamics play an important role in cell and animal physiology. An imbalance in the control of mitochondrial fusion and fission dramatically alters overall mitochondrial morphology [11,12]. This balance is regulated by a family of dynamin-related GTPases that exert opposing effects. In mammals, OPA1 and mitofusins are required for mitochondria fusion. In contrast, Drp-1 regulates mitochondrial fission [10,12]. Recent evidence indicates that OPA1 release participates in the rapid and complete release of cytochrome C in apoptotic cell death as well as controls apoptotic cristae remodeling [22,26]. In the present study, we found a small isoform of OPA1 (80 kDa) in the cytosolic fraction and two isoforms of OPA1 (90 and 80 kDa) in the mitochondrial fraction of the normal rat retina. In contrast, ischemic rat retina showed at least four isoforms of OPA1 in the cytosolic fraction at 6 h and 12 h. Interestingly, while both 90 and ~65 kDa isoforms of OPA1 were significantly increased in the cytosolic fraction, the large isoform of OPA1 (90 kDa) was significantly decreased in the mitochondrial fraction. These results indicate that ischemic damage following acute IOP elevation directly induces OPA1 release from mitochondria to the cytoplasm in the retina. Activation of rhomboid intramembrane protease (PARL) may explain the cleavage of OPA1 into truncated forms observed in the present study following ischemia [28]. OPA1 may be crucial for the anti-apoptotic effects of PARL because it maintains the bottleneck configuration of cristae and the comparmententalization of cytochrome C [25,28]. Thus, it is likely that the unexpected smaller molecular weight of OPA1 fragments presently observed might include the truncated forms of OPA1 that localize to in the intermembrane space or possibly one of the degradation products. Although OPA1 consists of at least five isoforms in other types of cells including HeLa cells [29-31], the precise functional role of the OPA1 isoforms that are released from mitochondria remains unknown. Thus, the potential proteolytic processing of OPA1 as well as the functional role of each OPA1 isoform in the cytosolic and mitochondrial fractions of the ischemic retina need to be further explored.

OPA1 is ubiquitously expressed in several tissues but is most abundant in the retina [15,17,18]. Pesch et al. [17] reported that the *OPA1* gene is expressed and its protein is present in RGCs and displaced amacrine cells in the normal rat retina. Further, we also reported that OPA1 protein is present in the amacrine cells and RGCs as well as horizontal cells of the normal rat retina [18]. Like OPA1, glutamate receptor subunits are present in horizontal cells, bipolar cells, amacrine cells, displaced amacrine cells, or RGCs in the rat retina [32-38]. It has been reported that many amacrine cells in the INL were rapidly and dramatically injured or killed by glutamate or NMDA and that displaced amacrine cells in the GCL also became swollen [38]. Thus, it is possible that glutamate receptor activation-mediated excitotoxicity may directly or indirectly contribute to neuronal cell death in the inner and outer retinal layer. Moreover, glutamate neurotoxicity is partly responsible for ischemic retinal damage [39-43]. Hence, it is possible that OPA1-positive cells in the rat retina may also have glutamate receptors.

In the present study, blockade of glutamate receptor activation prevented OPA1 release and apoptotic cell death as well as increased *OPA1* gene and total protein expression in ischemic rat retina. These results are good agreement with previous studies that down-regulation of *OPA1* causes mitochondrial fission, leading to cytochrome C release and apoptosis in HeLa cells, as well as induces aggregation of the mitochondrial network in purified RGCs [20-23] and that increased *OPA1* expression protects cells from apoptosis by preventing cytochrome C release and by stabilizing the shape of mitochondrial cristae [22,23]. Moreover, OPA1 deficiency in mouse models of ADOA impairs mitochondrial morphology, optic nerve structure, and visual function [44,45]. Together with these findings, our observations suggest that alteration of *OPA1* expression and distribution by glutamate receptor activation may signal in the neurodegenerative events in ischemic insult-related glaucomatous optic neuropathy.

Bax is a proapoptotic member of the Bcl-2 family that is essential for many pathways of apoptosis [46]. Bax directly interacts with the components forming the mitochondrial permeability transition pore (MPTP) that allow proteins to escape from the mitochondria into the cytosol to initiate apoptosis [47-49]. In the present study, we found that ischemia induced significant increases of *Bcl-2* and *Bax* mRNA expression in the retina at 12 h showing apoptotic cell death. Interestingly, blockade of glutamate receptor activation did not alter the increase of *Bax* expression but significantly increased *Bcl-2* expression at 12 h. Perhaps, this increased Bcl-2 may block the Bax-mediated MPTP formation that may cause OPA1 release from mitochondria in ischemic retina. These observations suggest that increased *OPA1* expression by blockade of glutamate receptor activation may contribute to retinal neuron survival in the presence of ischemic stress. Further studies will be needed to determine the mechanism of this effect and to clarify the relationships among glutamate receptor activation, OPA1 release, and increases of Bcl-2 and Bax following ischemic damage.

In summary, our findings demonstrated that glutamate receptor activation triggers OPA1 release in the early neurodegenerative events and that blockade of glutamate receptor activation prevents OPA1 release and apoptotic cell death as well as increases *OPA1* expression and total protein expression in the ischemic rat retina. This suggests that defined OPA1 distribution changes may be a factor in the apoptosis induced by glutamate receptor activation in ischemic retina. Moreover, Bcl-2 and Bax alterations may also contribute to these events though further study will be needed to clarify their relationship. Together, these results raise the possibility that OPA1 release induced by glutamate receptor activation contributes to mitochondrial dysfunction in the pathophysiology of pressure-related ischemic damage in glaucomatous retina.
